# BiotechLec: an interactive guide of commercial lectins for glycobiology and biomedical research applications

**DOI:** 10.1093/glycob/cwad034

**Published:** 2023-04-21

**Authors:** Boris Schnider, Francisco L Escudero, Anne Imberty, Frédérique Lisacek

**Affiliations:** Proteome Informatics Group, SIB Swiss Institute of Bioinformatics, route de Drize 7, Geneva CH-1227, Switzerland; Computer Science Department, University of Geneva, route de Drize 7, Geneva CH-1227, Switzerland; Proteome Informatics Group, SIB Swiss Institute of Bioinformatics, route de Drize 7, Geneva CH-1227, Switzerland; University Grenoble Alpes, CNRS, CERMAV, 601 rue de la chimie, Grenoble 38000, France; Proteome Informatics Group, SIB Swiss Institute of Bioinformatics, route de Drize 7, Geneva CH-1227, Switzerland; Computer Science Department, University of Geneva, route de Drize 7, Geneva CH-1227, Switzerland; Section of Biology, University of Geneva, route de Drize 7, Geneva CH-1227, Switzerland

**Keywords:** biotechnology, carbohydrate-binding, database, glycosylation, lectins

## Abstract

For decades, lectins have been used as probes in glycobiology and this usage has gradually spread to other domains of Life Science. Nowadays, researchers investigate glycan recognition with lectins in diverse biotechnology and clinical applications, addressing key questions regarding binding specificity. The latter is documented in scattered and heterogeneous sources, and this situation calls for a centralized and easy-access reference. To address this need, an on-line solution called BiotechLec (https://www.unilectin.eu/biotechlec) is proposed in a new section of UniLectin, a platform dedicated to lectin molecular knowledge.

## Introduction

Because of their specificity for glycans, lectins are widely used as biotechnological tools (Lis and Sharon 1986; Cummings et al. 2022). Their application extends further than the domain of glycobiology since lectins are effective markers in cell typing and as such, frequently used in immunology, cancer research, and clinical microbiology, to name only a few areas. One of the oldest usages of lectins is testing the agglutination and precipitation of glycoconjugates, cells, and membrane vesicle preparations. The first application of this was blood group typing by erythrocytes hemagglutination in 1953 (Morgan and Watkins 1953). Lectins provide further resources: they can be immobilized and utilized for the purification of glycoproteins, and they are also classically employed to characterize cell-surface glycoconjugates through histochemistry or flow cytometry with cell sorting. For example, sorting residual human pluripotent stem cells from cell cultures is achieved with BC2L-CNt, a bacterial lectin (Haramoto et al. 2020). Lectins are handy in enzymology for assaying specific glycosyltransferases and glycosidases. In recent years, they have been immobilized on microarrays or other biosensors to characterize the glycan moiety of glycoproteins, cell extracts, or whole cells (Ribeiro and Mahal 2013). They are also used for cell-type specific intracellular delivery of cargo, for example, by conjugation to nanodiamonds in brain cell imaging (Ghanimi Fard et al. 2022).

Lectins are often preferred to other glycan-binding proteins, such as antibodies offering higher specificity but usually costlier. Compared with carbohydrate-binding modules (CBMs), their multi-valency makes them effective reagents for affinity chromatography of glycoproteins and glycans and histology biomarkers. Traditionally, lectins made available in the catalogues of (bio) chemicals have been purified from seeds and plant materials. However, an increasing number is now obtained from fungi and animals, as well as bacteria and viruses. Although most of the commercial lectins are purified from natural sources, they can also be produced recombinantly, which offers advantages of purity and reproducibility and opens other engineering possibilities.

Such a wide and diverse range of applications, together with a growing collection of solutions, may become a source of confusion for scientists addressing specific carbohydrate-binding related issues. The purpose of the present note is therefore to describe a means of supporting researchers in selecting the most suitable lectin(s) to solve the corresponding carbohydrate-binding questions.

## What is BiotechLec?

**Fig. 1:**
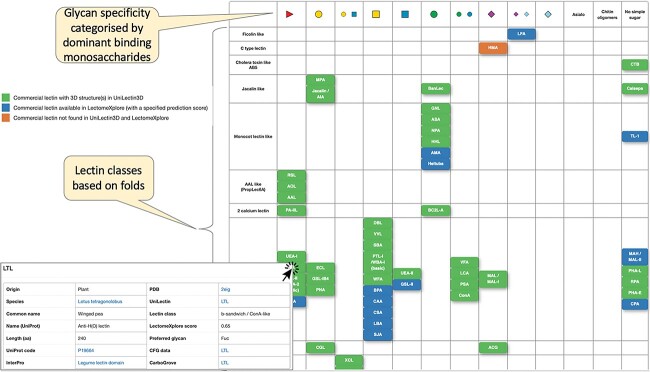
Rows of the table are lectin fold classes defined in UniLectin. Columns are defined by monosaccharide ligands best characterizing the lectin specificity and pictured with the SNFG notation (https://www.ncbi.nlm.nih.gov/glycans/snfg.html). A table cell is filled when the fold of a commercial lectin matches a given glycan specificity. The distribution is uneven. For example, 11 lectins adopting the L-type legume fold bind to GalNAc. The color code is green for curated and blue for predicted information. Orange is used in the few cases when no sequence data are available. Each item can be clicked on to prompt a summary table with various links to further protein details and glycan-binding information.

In 2018, we launched the UniLectin portal (unilectin.eu) with the ultimate aim of bringing the broadest coverage of lectin molecular knowledge. UniLectin is dedicated to the classification, curation, and prediction of lectins (Imberty et al. 2021). The platform is composed of several modules, starting with Unilectin3D, a curated database with >2,400 3D-structures, which sets the basis for classification (Bonnardel et al. 2019b). The other modules provide information on predicted lectomes (i) from all available translated genomes—LectomeXplore (Bonnardel et al. 2021), (ii) from specific origin—MycoLec (Lebreton et al. 2021) and (iii) reflecting structural families—PropLec and TrefLec (Bonnardel et al. 2019a; Notova et al. 2022). Here, we introduce the BiotechLec module (https://www.unilectin.eu/biotechlec) that centralizes information about lectins that are commercially available and classically used for research. A recent review summarizing the activity of 57 of such lectins (Bojar et al. 2022) motivated the design of BiotechLec as an interactive complement. A larger screen of on-line catalogues (Vector Laboratories, Glycodiag, Elicityl and Wako companies) led to identify 77 lectins that were first organized in a tabular format, reflecting the classification adopted across the whole UniLectin platform and their main glycan specificity (Fig. 1).

Although a large majority is purified from plants (66%), the table shows that fungal (13%) and bacterial (10%) origins are also represented. Animal lectins are less commonly used and they originate mostly from invertebrates (snail, slug, and lobster) or fish (eel). The consequence is that many of the listed lectins belong to the legume lectin class. Conversely, some classes are not represented such as I-type lectins, mainly found in mammals so far. It should be noted that some human lectins are available commercially, but they are not included in the database since they are not classically used for purification or diagnostic purposes.

Glycan specificity is represented by the monosaccharide ligands that are used for eluting the lectin, as listed in catalog or literature, even though the specificities may involve more complex glycan sequences. Monosaccharides are depicted in the widely adopted Symbol Nomenclature for Glycans (SNFG) (Neelamegham et al. 2022). Galactose and GalNAc are overrepresented in the specificity, while sialic acids (NeuAc and NeuGc) have only a few binding representatives. The double Gal/GlcNAc specificity from two mushroom lectins (XCL and ABA) is explained by the occurrence of two distinct binding sites with different specificities on each monomer in the fungal fruit-body lectin class (Carrizo et al. 2005).

## How to explore BiotechLec

A three-color code was adopted to characterize each item, symbolized by the lectin abbreviated name, in the table. A green box corresponds to a curated UniLectin entry that includes 3D structural information, while a blue box points to entries with amino acid sequence information and prediction based on similarity (the quality of this prediction is evaluated with a score calculated by the LectomeXplore engine (Bonnardel et al. 2021)). Three orange boxes are lectins lacking sequence information and therefore not linked to any UniLectin entry (all UniLectin entries are associated with an amino acid sequence and a link to a protein database). Each colored box of the BiotechLec dataset is clickable. Information is provided in a small pop-up table. In the first column, the biological origin of the lectin is described through the species name (with link to the NCBI taxonomy browser) and the common name. Information on the protein includes the UniProt name and accession number (www.uniprot.org), amino acid sequence length, and reference to the InterPro database of protein families (www.ebi.ac.uk/interpro). Even for the small number of lectins lacking a protein sequence (not published or deposited), a UniProt reference is proposed nonetheless. It is chosen as the most likely one, considering the information available and not dismissing the possibility of isolectins. Corrections and additions are easily made, and feedback on entries is welcome. The second column of the pop-up table contains information from (a) the Protein Data Bank (www.rcsb.org), when the 3D structure is available, (b) the Unilectin portal and associated classification, and (c) websites dedicated to the analysis of lectin specificity. The Unilectin entry directs either to the UniLectin3D module of curated lectins for which structural data are available or to the LectomeXplore module of predicted lectins displaying sequence alignment with a class representative. When several crystal structures are available, the one with the most representative ligand i.e. a natural ligand with documented high affinity, has been selected, but all other structures are available from UniLectin3D through the Unilectin link. It should be noted that when no crystal structure is available, a model generated by AlphaFold is available in the corresponding UniProt entry. This is of interest, for example, for tomato lectin (LEA), which is widely used for labeling vasculature but could never be crystallized, and appear to be modelled with the occurrence of five hevein domains. Finally, glycan array data are available, either as raw data from the Consortium for Functional Genomics (http://www.functionalglycomics.org) or as analyzed results from CarboGrove (carbogrove.org) (Klamer et al. 2022). For both websites, one representative entry was selected to provide a clear view of oligosaccharide specificity, with concentrations low enough to avoid cross-reactivity and high enough for strong signal/noise ratio.

## Conclusion

BiotechLec was created to guide experimentalists in their choice for the right lectin depending on the aim of the assays that may be constrained by size, structure, multivalency, and/or fine specificity. It should also help rationalizing results of assays built using commercial lectins. It is not intended as a full fledge database, but a set of shortcuts to key information regarding lectins commonly used as probes. Other databases provide information on structure, sequence, and specificity of an overlapping set of lectins, such as the Lectin Frontier Database containing ~400 entries (https://acgg.asia/lfdb2/index) and GlyCosmos Lectins (https://glycosmos.org/lectins) that combines several sources including UniProt and UniLectin into roughly 2,300 entries. These resources are general-purpose, in contrast with BioTechLec, which has a user-friendly interface to quickly access information on commercial lectins. This guide was also designed to easily account for regular updates reflecting, as mentioned above, researchers’ comments and feedback on testing the listed lectins or newly published information. It can equally as easily accommodate the addition of new entries.

## Data Availability

All data are available in the database https://www.unilectin.eu/biotechlec/.
